# Towards interpretable, medically grounded, EMR-based risk prediction models

**DOI:** 10.1038/s41598-022-13504-7

**Published:** 2022-06-15

**Authors:** Isabell Twick, Guy Zahavi, Haggai Benvenisti, Ronya Rubinstein, Michael S. Woods, Haim Berkenstadt, Aviram Nissan, Enes Hosgor, Dan Assaf

**Affiliations:** 1 Caresyntax GmbH, Komturstraße 18A, 12099 Berlin, Germany; 2 Department of General and Oncological Surgery – Surgery C, The Chaim Sheba Medical Center, Ramat Gan, Israel; 3 Department of Anesthesiology, The Chaim Sheba Medical Center, Ramat Gan, Israel

**Keywords:** Risk factors, Machine learning, Predictive medicine

## Abstract

Machine-learning based risk prediction models have the potential to improve patient outcomes by assessing risk more accurately than clinicians. Significant additional value lies in these models providing feedback about the factors that amplify an individual patient’s risk. Identification of risk factors enables more informed decisions on interventions to mitigate or ameliorate modifiable factors. For these reasons, risk prediction models must be explainable and grounded on medical knowledge. Current machine learning-based risk prediction models are frequently ‘black-box’ models whose inner workings cannot be understood easily, making it difficult to define risk drivers. Since machine learning models follow patterns in the data rather than looking for medically relevant relationships, possible risk factors identified by these models do not necessarily translate into actionable insights for clinicians. Here, we use the example of risk assessment for postoperative complications to demonstrate how explainable and medically grounded risk prediction models can be developed. Pre- and postoperative risk prediction models are trained based on clinically relevant inputs extracted from electronic medical record data. We show that these models have similar predictive performance as models that incorporate a wider range of inputs and explain the models’ decision-making process by visualizing how different model inputs and their values affect the models’ predictions.

## Introduction

Each year, around 310 million patients undergo surgery worldwide, 17% of which develop one or more postoperative complications^1–3^. Adverse outcomes are not only a burden to patients and their families—causing reduced quality of life, morbidity, and mortality—they also strain hospital resources and amplify healthcare costs^4–6^. Costs for additional treatments and surgeries, extended hospital stays, and readmissions associated with the occurrence of any complication are estimated to be 1.5 to fourfold higher^7,8^. Accurate patient risk assessment and mitigation are therefore essential to reduce complications and associated patient burden and costs.

Risk assessment for postoperative complications can be beneficial at different stages during the surgical encounter. Preoperative risk assessment is essential for an informed discussion with the patient about the risks and benefits of surgery and helps identify patients that may benefit from preventive preoperative and intraoperative strategies. A postoperative risk update that incorporates intraoperative data about potential complications and irregularities during surgery can support postoperative care decisions , including patients monitoring, radiologic studies and prophylactic measures.

Risk for postoperative complications arises from many factors, including a patient’s health status prior to surgery, the type of surgical procedure performed, the surgeon’s skill, and the patient’s physical capacity to withstand surgical stress and anesthesia associated with the procedure. Although risk factors have been identified for various postoperative complications^9,10^ assessing a patient’s individual risk is still a challenge for physicians. Precise and timely risk prediction proves difficult as it requires the interpretation and combination of large amounts of clinical information.

To support the physicians’ decision process of assessing patients’ risk for postoperative complications, a variety of risk scores have been developed that differ with respect to the underlying data, the adverse outcome, and identified risk factors^11^. While rather rudimentary risk scores such as P-POSSUM, APGAR, and ASA are still widely used thanks to their simplicity^12–14^ the favored risk score of many physicians is the universal National Surgical Quality Improvement Program (NSQIP) risk calculator developed by the American College of Surgeons^9,15^. It is based on a logistic regression model with 21 input variables covering patient characteristics and type of the procedure. Trained on the largest outcome dataset of more than 5 million records collected from 855 hospitals and more than 1,800 surgical procedures, the NSQIP score performs preoperative risk assessment for 16 general and two procedure-specific 30-day adverse outcomes (https://riskcalculator.facs.org).

While the performance of the NSQIP risk calculator as a ‘universal’ risk assessment tool has been confirmed by several studies^15,16^ it has some major drawbacks which limit its usage: Firstly, poor usability. The risk calculator is outside the day-to-day workflow of surgeons and their staff, requiring manual data input into a web interface. It does not leverage available electronic medical record (EMR) data, adding an unnecessary burden for physicians. Secondly, it acts as a black-box model, only displaying a patients’ risk without any transparency on which factors influence the prediction and to what effect. Thirdly, it only performs preoperative risk assessment, not accounting for intraoperative data that may improve the risk estimate and could therefore support postoperative care decisions. And finally, it only considers linear relationships between input variables while, in reality, interactions between risk factors and adverse outcomes can be and often are nonlinear^17^.

The recently developed *MySurgeryRisk* platform illustrates how EMR data can be leveraged for risk prediction. *MySurgeryRisk* is an automated predictive analytics framework that utilizes clinical EMR data to forecast patient-level risk scores for eight major postoperative complications and death^18^. Generalized additive models (adjusted for nonlinearity) were trained on a sample of 51 k surgical patients from a single medical center. The machine learning models achieved good predictive performance and discrimination of high and low-risk patients. Through the integration with the EMR, *MySurgeryRisk* automatically assesses the risk of a patient undergoing surgery and can thus be seamlessly incorporated into a physician’s workflow^17^. Since its implementation, *MySurgeryRisk* has further been extended to include postoperative risk assessment^19^. By incorporating intraoperative data, postoperative risk prediction substantially outperformed preoperative models. Although *MySurgeryRisk* displays the most important features impacting a patient's risk score, the model’s inner workings cannot be interpreted easily, nor can clinical relevance be guaranteed.

The *POTTER* risk calculator demonstrates an example of a nonlinear risk prediction model that does not sacrifice model interpretability. It is based on Optimal Classification Trees, a type of nonlinear decision tree that enable the model’s decisions to be tracked by following down the decision nodes in the tree structure^20^. *POTTER*’s preoperative risk prediction models were trained on data of 400 k emergency surgery patients collected as part of the American College of Surgeons NSQIP^20^. It predicts postoperative mortality, morbidity, and 18 specific complications. While not integrated with the EMR, a smartphone application was developed that physicians can use to derive a patient’s risk prediction. Although POTTER is a great advancement towards more explainable risk prediction models, the complexity of the tree structure with hundreds of input variables, tree depths in the range of dozens and hundreds of decision nodes make it difficult to grasp how the model comes up with a patient’s risk score. Besides, while Optimal Classification Trees perform better than other decision trees, they are still outperformed by more complex models such as random forest algorithms^20^.

Here, we aim to follow up on the advances made by *MySurgeryRisk* and *POTTER* by developing interpretable risk prediction models that are based on EMR data without compromising on predictive performance.

## Methods

### Ethical approval

The institutional review board—the Tel Hashomer Helsinki Committee—approved data access and study design and waived the need to obtain informed consent (SMC-6411-19). All methods were performed in accordance with the relevant guidelines and regulations.

### Data

#### Dataset

The data sample contained all patients undergoing surgery between September 2017 and September 2019 in the General and Oncology Surgery department at Sheba Medical Center, Israel. In total, 3440 patients undergoing 4004 surgeries were included in the dataset.

#### Outcomes

Postoperative surgical complications were prospectively captured through a manual review performed by the medical staff. The labeling methodology was based on the Clavien Dindo complication scale.

#### Features

Descriptive features were calculated from EMR data collected at hospital admission, during the preoperative hospital stay, and intraoperatively. In total, 969 features were calculated, of which 741 were used for preoperative and all 969 for postoperative risk prediction. Preoperative features included patient characteristics such as demographics, comorbidities, alcohol and smoking habits, prescribed drugs, past procedures, and preoperative data collected in the hospital including vitals, lab and microbiology, medication in the department and anesthesiologist checks. For postoperative models, intraoperative data such as surgery specifics, administered medications, vital and anesthesia measurements, and information about blood loss and transfusion were also included. Supplementary “*List of features”* provides an overview of all features; supplementary “*Feature Creation*” explains data preprocessing and feature calculation steps.

#### Selection of risk factors

Risk factors for surgical site infection (SSI) and anastomosis leak complications were identified based on a selective literature review. Criteria for risk factor selection were consistent identification of the factor as increasing the risk for the respective complication and the factor being available in our EMR dataset. SSI risk factors relied largely on systematic reviews^21,22^ and a large multicenter study based on data from the National Surgical Quality Program^23,24^. For leak risk factors we utilized a systematic review^25^ and a large multicenter prospective study^26^.

### Machine learning modeling

#### Sample size

The dataset sample of 4004 surgeries was split into a 70% training and 30% testing set. Random splitting was performed based on patient id to ensure that all surgeries of a given person were contained in the same set.

#### Modeling

Non-linear tree-based gradient boosting classifiers were trained using the *CatBoost* implementation^27^. Missing values were imputed as the minimum values (less than all other values) for the feature to guarantee that a split is able to separate missing values from all other values. Area under the curve (AUC) was chosen as the evaluation metric, and overfitting was controlled by early stopping after 50 iterations following the optimal metric value was reached. Recursive backward feature elimination was performed to determine the lowest number of features without sacrificing model performance.

Best performing models were chosen based on the highest average AUC of fivefold cross-validation within the training set. Models were refitted on the whole training set, and performance was checked on the hold-out test set (Supplementary Table S7). Final models were calibrated to return probabilities using logistic regression trained on the model outputs from the train set.

#### Model performance

Overall model performance was assessed based on the area under the curve (AUC) of the receiver operating characteristic curve (the sensitivity vs. (1-specificity) plot). The AUC represents the probability that a randomly chosen positive example has a higher predicted probability score than a randomly chosen negative example. It, therefore, measures the discrimination of positive and negative events^28^. An AUC of 1 denotes perfect discrimination; 0.5 is no better than chance.

Since the output of the models is a risk probability that ranges from 0 to 1, a probability threshold was chosen to separate patients into a high and a low-risk group. Probability thresholds were determined by the highest Matthew Correlation Coefficient (MCC, Supplementary Table S8) or an 80% sensitivity of the model (Table 1). MCC is a measure of association between the observed and predicted binary classifications. It is regarded as the best measure to describe all four confusion matrix categories (true positives, false positives, true negatives, false negatives), superior to the commonly utilized F1 measure, which ignores correctly classified negative samples^29^. The MCC returns values between − 1 and + 1, whereas a coefficient of + 1 represents a perfect prediction, 0 is no better than random chance, and − 1 indicates complete disagreement. For easier comparison between preoperative and postoperative model performances, we additionally chose the probability threshold based on 80% sensitivity. In this case, the models detect 80% of the patients that develop an SSI or leak. Based on both types of probability thresholds (MCC or 80% sensitivity), we report other statistics including sensitivity, specificity, positive predicted value (PPV), negative predicted value (NPV), and MCC.

**Table 1:** Overview of Model Performances according to 80% sensitivity.

Model	Pre/Post	Features	AUC	Threshold	MCC	Sensitivity	Specificity	PPV	NPV
**SSI models**									
Naïve Gradient Boosting	Pre	8	0.759	0.046	0.236	0.805	0.540	0.106	0.976
	Post	56	0.857	0.041	0.308	0.805	0.761	0.185	0.983
Literature-based Gradient Boosting	Pre	12	0.762	0.044	0.208	0.805	0.615	0.124	0.979
	Post	15	0.853	0.049	0.317	0.805	0.770	0.191	0.983
**Leak models**									
Naïve Gradient Boosting	Pre	60	0.775	0.033	0.134	0.795	0.582	0.059	0.988
	Post	82	0.855	0.031	0.222	0.795	0.758	0.098	0.981
Literature-based Gradient Boosting	Pre	8	0.793	0.031	0.160	0.795	0.644	0.069	0.990
	Post	10	0.861	0.034	0.233	0.795	0.774	0.104	0.991

Table depicts model performance of all pre- and postoperative SSI and leak models developed as part of this study. *Naïve Gradient boosting* refers to gradient boosting models where all pre-calculated features were considered, and the type and number of features were selected based on recursive feature elimination. *Literature-based Gradient Boosting* are gradient boosting models developed based on features reported in the literature combined with recursive feature elimination to reduce the number of features included in the models. Table contains AUC (area under the ROC curve) computed for the test set as well as specificity, PPV (positive predicted value), NPV (negative predicted value), and MCC (Matthew Correlation Coefficient) with respect to the probability threshold chosen to achieve 80% sensitivity.

### Model explanation

#### Shap values

Shapley Additive Explanations (SHAP), a model-agnostic explanation technique derived from cooperative game theory, was used to interpret the predictions of the gradient boosting machine learning models^30^. SHAP values explain the contribution of a feature and its value to the prediction. SHAP values have the property that they sum up to the difference between the average model output and the model output of the respective sample. In simple terms, SHAP values are derived by comparing the model’s predictions with and without the feature^30^. A positive SHAP value indicates that the corresponding feature/value pair increases the risk of a complication; a negative SHAP value indicates that the pair lowers the risk. The magnitude of SHAP values represents how much the feature/value pair contribute towards the models’ prediction. The importance of a feature was determined by summing the absolute SHAP values of the feature for all samples.

## Results

### Patient population characteristics and outcomes

The dataset contained 4004 surgeries performed on 3440 patients in a two-year period at the Sheba General and Oncology Surgery Department in Tel Aviv, Israel (Supplementary Table S5). Patients undergoing surgery had a median age of 55 (25th–75th percentiles 40–67 years), with 55% being female. The most frequent comorbidities were neoplasms (19%), circulatory (8.9%), metabolic (8.4%), and digestive (7.4%) diseases. Accordingly, a large subset of patients took medications including cardiovascular (35.1%), metabolism (33.1%), blood (21.6%), and nervous system drugs (21.6%). Surgery types from high to low volume included hernia (14.4%), gastroesophageal (13.2%), colorectal (12.8), biliary tract (11.2%), breast (10%), and diagnostic (7.3%). Less than half of the surgeries were laparoscopic (46.9%), and around one quarter were urgent procedures (25.3%).

Surgical site infections (SSIs) were the most prevalent postoperative complication, which developed in 5.8% of the cases, followed by leaks with an incidence of 3.4% (Supplementary Tables S5 and S6). Among leak eligible cases including surgeries classified as gastroesophageal, biliary tract, colorectal or small bowel, leaks occurred in 5.6% of the cases. SSIs composed of superficial, deep, and organ-space infections occurred most frequently in colorectal (18.4%), small bowel (17.4%), abdomen/ retroperitoneum (10.6%) as well as diagnostic cases (7.2%). Leaks which included gastrointestinal, biliary, pancreatic, and anastomosis leaks prevailed in small bowel (12.4%), colorectal (9.1%), and abdomen/retroperitoneum (6.7%) surgeries.

### Risk prediction models based on electric medical records

Firstly, we developed pre- and postoperative risk prediction models for SSI and leak that incorporated all features available at the time of prediction, at the start or end of the surgery, respectively. The predictive performance for SSI and leak models were higher for postoperative than for preoperative risk models (Table 1). Risk prediction of SSI in the test cohort increased from an AUC of 0.76 preoperatively to 0.86 postoperatively. Similarly, the prediction of leaks increased from an AUC of 0.78 preoperatively to 0.86 postoperatively. To separate patients into high and low-risk groups, a probability threshold was chosen using maximal MCC (Supplementary Table S8) and, for easier comparison between preoperative and postoperative models, based on 80% sensitivity (Table 1). At 80% sensitivity, both pre- and postoperative models achieved high NPVs ranging from 0.98 to 0.99. Preoperative PPVs of 11% for SSI and 6% for leaks increased to 19% and 10% postoperatively. Comparisons of model performance between train and test sets can be found in Supplementary Table S7; performance metrics of leak models based on leak relevant cases are depicted in Supplementary Table S9.

While the preoperative SSI model relied on a limited number of eight features, postoperative SSI and leak models included tens of features (56 features for postoperative SSI, 60 and 82 features for pre- and postoperative leak models, respectively). The most important features for preoperative SSI prediction were previous colorectal surgery, the patient’s age, and the most recent preoperative C-reactive protein (CRP) results (Supplementary Fig. S1). Postoperative SSI prediction relied mainly on the surgical approach (open versus laparoscopic), the number of procedures performed, and the number of analgesic medications administered during surgery (Supplementary Fig. S1). Preoperative leak prediction was mainly influenced by features such as BMI, number of past procedures, and the average prothrombin time in the three preoperative days (Supplementary Fig. S2). Postoperatively, the most important features were placement of an arterial line, operation duration, and the intraoperative ratio of low end-tidal CO2 (Supplementary Fig. S2).

### Risk factors identified through literature review

The developed risk models rely on a large number of features, also including data sources that have not been identified as risk factors in the literature before, such as prothrombin, end-tidal CO2, or analgesic drugs. We were interested in whether models based on previously identified risk factors would perform equally well as those incorporating all the input data. We, therefore, performed a selective literature search to identify the most common risk factors for postoperative SSI and leak occurrence. Importantly, our research was solely focused on risk factors that are standardly collected within the EMR. Thus, some well-known risk factors that cannot be captured through EMR records were not considered.

Due to their high prevalence, patient burden, and cost risk factors for SSI have been studied extensively. Some of the identified risk factors increase the risk of SSI in general; others are specific to certain surgical procedures^21^. General risk factors include patient-related factors such as patient demographics (e.g. gender and age), a patient’s health status (e.g. BMI and ASA score) as well as lifestyle habits (e.g. smoking and alcohol consumption)^21,22^. Comorbidities such as diabetes and malignant cancers likewise increase SSI risk , as do preoperative hypoalbuminemia, steroid intake and remote infections^21,22^. Other general risk factors are related to the patient’s preparation for surgery and the surgical procedure itself. These include the length of the preoperative hospital stay, antibiotic prophylaxis, the type of surgery, and whether the surgery is urgent^21–23^. Intraoperative risk factors are blood loss and transfusion, the duration of the surgery, and wound type (clean, contaminated, etc.)^21^.

Although leaks are a feared complication in colorectal surgery and have been the focus of many studies, risk factors are less well understood^31^. Many of the identified risk factors are equal to those identified for SSI. These include gender, age, BMI, ASA score, smoking, alcohol consumption, diabetes, corticosteroid intake, preoperative hypoalbuminemia, antibiotic prophylaxis, urgent surgery, blood loss and transfusion, surgery duration, open surgery, and complexity of the procedure^25^. Additionally, intake of anticoagulants, preoperatively low total serum protein, and intraoperative complications are known risk factors for leaks^26^.

Many identified risk factors also increased the risk of developing SSI and leak in our dataset. Table 2 lists the risk factors for SSI and leaks and their associated odds ratios. For SSI, all risk factors apart from smoking had an odds ratio (OR) larger than one and thus increased the risk of SSI. Largest odds ratios were found for antibiotics administered up to 2 h before surgery (OR = 5), blood transfusion (OR = 4.2), surgery duration longer than 120 min (OR = 5.5), intestinal procedure (OR = 7.6), open surgery (OR = 8.6), and more than one intervention (OR = 5.4). For leaks, total serum protein < 4 g/dL (OR = 6), blood transfusion (OR = 6.1), surgery duration longer than 120 min (OR = 6.3), open surgery (OR = 5.2), and intestinal procedure (OR = 6.3) were associated with the largest odds ratios. Smoking, alcohol consumption, corticosteroid intake, and diabetes disease did not increase the risk of leaks (OR < = 1). Information on the procedure type was not available preoperatively since planned procedures were frequently not entered into the EMR. As a proxy for the performed procedure, we included the procedure type of a past surgery in the analysis. Since our data sample was collected in an oncology department, a large fraction of surgeries was cancer surgery, requiring multiple procedures of the same type if cancer spreads, reoccurs or has associated postoperative complications. For example, some procedures, such as colostomies, frequently need reoperations at the same location, so do surgeries that result in complications such as deep/organ SSI or leaking bowel connections. A past intestinal procedure had an increased odds ratio for SSI (OR = 4.9) and leaks (OR = 4.8).

**Table 2:** Risk factors, odds ratios and literature references for SSI and leaks.

Risk factor	SSI		Leaks	
	Odds ratio	References	Odds ratio	References
**Gender**				
Male	1.1	^21,22^	1.3	^25,50^
**Age**				
> 50	3.6	^22,23^	2.7	^25,50^
> 60	2.3		2.2	
**BMI**				
< 20 kg/m^2^	1.4	^21,22^	1.8	^25,26,50^
> 35 kg/m^2^	0.4		0.3	
**ASA score**				
≥ 3	2.4	^21–23^	2.3	^25^
≥ 4	3.3		3.2	
**Lifestyle habits**				
Smoking	0.6	^21–23^	1	^25,50^
Alcohol use	2		1	
**Medication**				
Corticosteroids	1.2	^21,23^	0.7	^25,26,50^
Anticoagulants	n.a		1.5	
**Comorbidities**				
Diabetes Type 1 or 2	1.7	^21–23^	0.6	^25^
**Labs**				
Albumin < 3.5 g/dL	3.2	^23^	3.7	^25,26,50^
Serum total protein < 4 g/dL	n.a		6	
Serum total protein > 8 g/dL	n.a		0.4	
**Prior infection**				
Microbiology test performed	2.8	^21,22^	n.a	n.a
Microbiology test positive	3.4			
**Antibiotics before incision**				
0 to 24 h before surgery	1.9	^21^	1.8	^25^
0 to 2 h before surgery	5		3.3	
**Length of preoperative stay**				
> 1 day	2.1	^21,22^	n.a	n.a
> 5 days	2.6			
> 7 days	3.4			
**Urgent procedure**				
Emergency / trauma	1.8	^21,23^	1.1	^25,26^
**Past procedure type**				
Intestinal	4.9	Procedure type^21,23^	4.8	Site of anastomosis^25,50^
**Blood**				
Loss ≥ 00c	3.3	^21,25^	2.6	^25,50^
Transfusion	4.2		6.1	
**Intraoperative complications**				
Complications	n.a	n.a	1.1	^26^
**Operation time**				
> 120 min	5.5	^21,22^	6.3	^25^
> 180 min	5.5		6.7	
> 240 min	4.4		7.8	
**Procedure type**				
Intestinal	7.6	^21,23^	6.3	Site of anastomosis^25,50^
**Surgical approach**				
Open	8.6	^24^	5.2	^25^
**Multiple interventions**				
Number of procedures > 1	5.4	Measure of complexity^21,22^	3.7	Measure of complexity^26^

Table shows risk factor for SSI and leaks identified based on a selective literature search together with their associated odds ratios calculated on the data set. *Past procedure type* was added as a preoperative indicator for the performed surgery as *procedure type* was only available postoperatively. n.a. (not applicable), the feature has not been identified as a risk factor.

### Literature-Informed Risk Prediction Model

Following the literature review, we aimed to test how machine learning models that solely rely on known risk factors perform compared to those that include all data inputs. For preoperative models, we included all preoperative risk factors of the respective complication, SSI or leak, listed in Table 2. For postoperative models, we combined pre- and intraoperative risk factors. As before we utilized gradient boosting models combined with recursive feature elimination to gain best performing models while limiting the number of input features. To explain how different features affect the model’s predictions, we used SHAP values.

**Figure 1:**
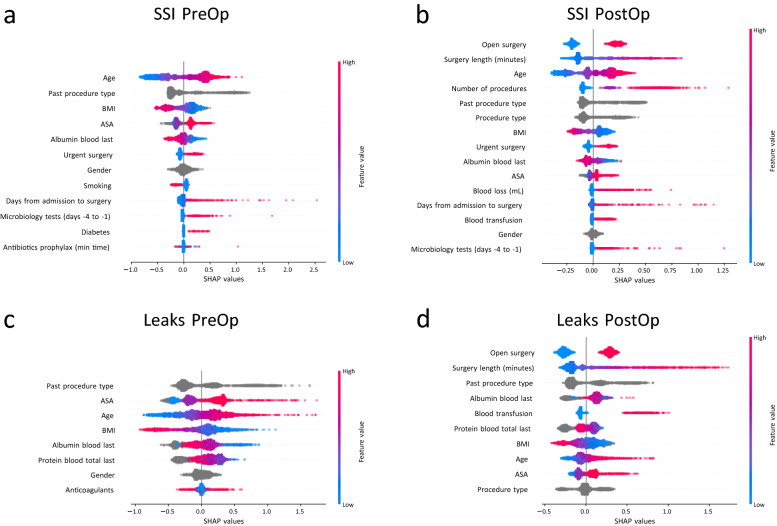
Literature-Informed Risk Prediction Models Figure shows SHAP summary plots displaying the effect of a feature and its value on the prediction. The y-axis of each plot contains the features included in the model sorted from the most (top) to least (bottom) important. The x-axis depicts the SHAP value. Each point refers to a SHAP value associated with a value of a certain feature. The color of the point displays whether the feature value is high (pink) or low (blue). Grey-colored points are categorical and missing values. Example: Age in subplot A: Older ages (red-colored points) increase the risk of SSI (SHAP value 0.5 to 1), lower ages (blue-colored points) reduce the risk (SHAP values − 0.9 to − 0.5). (**A**) Preoperative SSI model (**B**) Postoperative SSI model (**C**) Preoperative Leak model (**D**) Postoperative Leak model.

**Figure 2:**
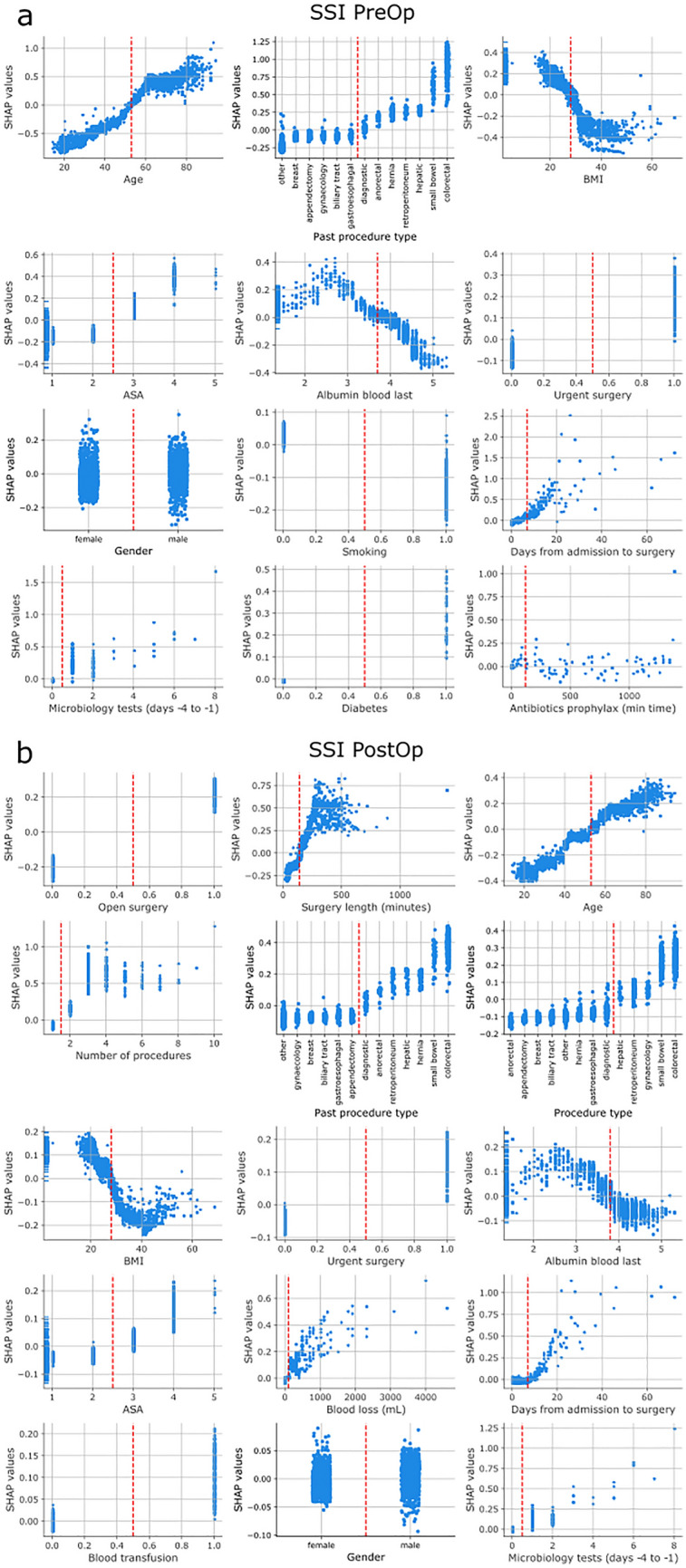
Impact of features on pre- and postoperative SSI risk predictions Subplots show how a given feature affects the risk prediction of the model. X-axes depict the values of the feature, y-axes the associated SHAP values. Missing values are represented on the far left of the x-axis. A positive SHAP value indicates that the value of the feature increases the overall risk, a negative SHAP values lowers risk of postoperative SSI. The larger the magnitude of the SHAP value the more it affects the risk prediction. Red vertical lines indicate inflection points were the value of the feature switches from having a negative to positive (or vice versa) impact on the risk prediction. The arrangement of the subplots follows the order of importance of the features for the model predictions. Importance was calculated by summing the absolute SHAP values of the feature of all samples. (**a**) Preoperative SSI risk models. Red vertical lines depict: Age = 53 years, BMI = 28 kg / m2, Albumin blood = 3.8 g/dL, Days from admission to surgery = 7 days, Microbiology test (on days − 4 to − 1 before surgery) = 1, antibiotics administered before surgery = 120 min. (**b**) Postoperative SSI risk models. Red vertical lines depict: Surgery length = 140 min, Age = 53 years, BMI = 28 kg / m2, Albumin blood = 3.8 g/dL, blood loss = 100 mL, Days from admission to surgery = 7 days, Microbiology test (on days − 4 to − 1 before surgery) = 1.

**Figure 3:**
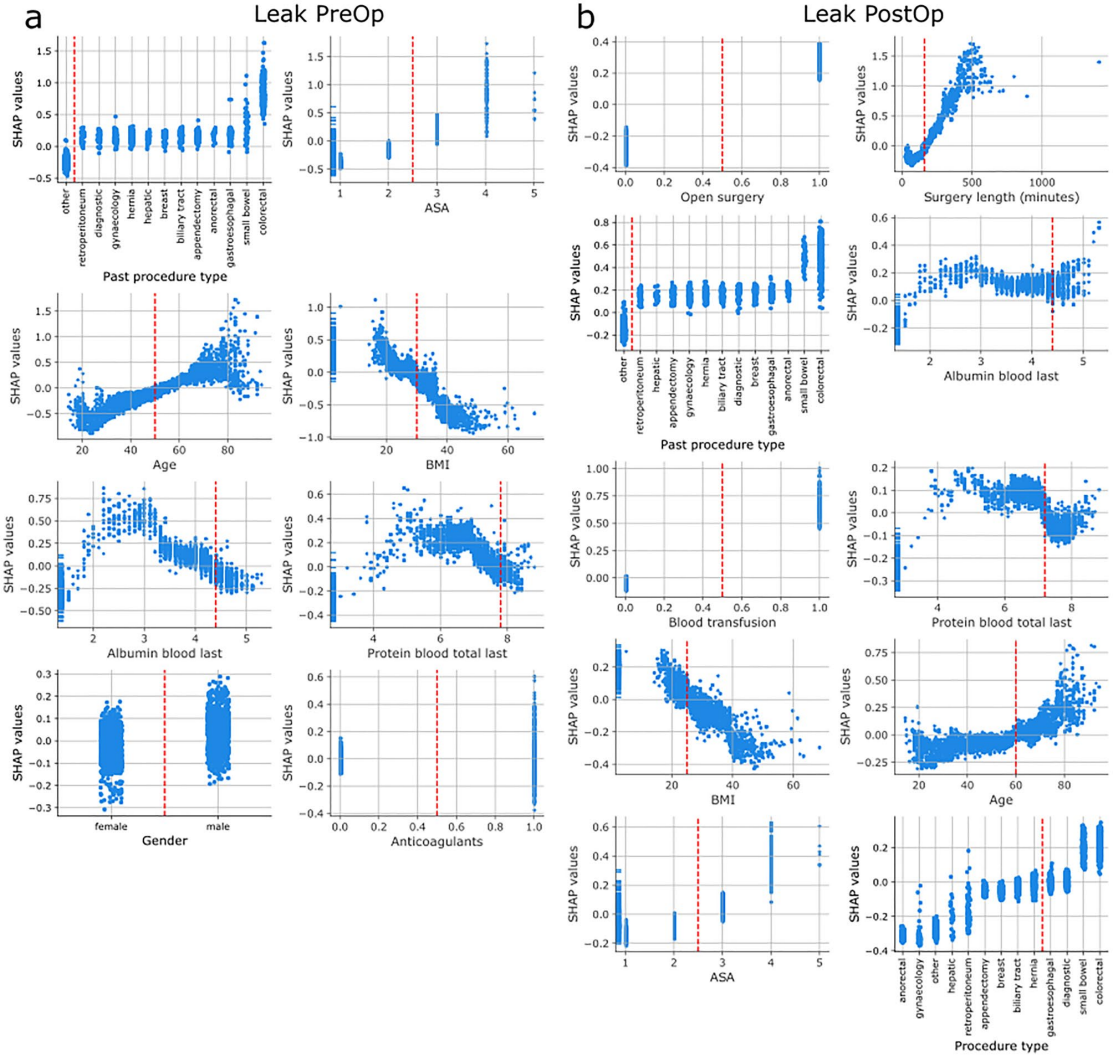
Impact of features of pre- and postoperative leaks risk predictions Subplots show how a given feature affects the risk prediction of the model. X-axes depict the values of the feature, y-axes the associated SHAP values. Missing values are represented on the far left of the x-axis. A positive SHAP value indicates that the value of the feature increases the overall risk, a negative SHAP values lowers risk of postoperative leaks. The larger the magnitude of the SHAP value the more it affects the risk prediction. Red vertical lines indicate inflection points were the value of the feature switches from having a negative to positive (or vice versa) impact on the risk prediction. The arrangement of the subplots follows the order of importance of the features for the model predictions. (**a**) Preoperative leak risk models. Red vertical lines depict: Age = 50 years, BMI = 30 kg / m2, Albumin blood = 4.4 g/dL, Protein blood total: 7.8 g/dL (**b**) Postoperative SSI risk models. Red vertical lines depict: Surgery length = 160 min, Albumin blood = 4.4 g/dL, Protein blood total = 7.2 g/dL, BMI = 25 kg / m2, Age = 60 years.

Predictive performance of these literature-informed models was comparable to the models developed based on all available inputs (Table 1 compares *Literature-based Gradient Boosting* with *Naïve Gradient Boosting* models, see Supplementary Table S7 for comparison of model performances between train and test sets and Supplementary Table S9 for performance metrics of leak models based on leak relevant cases). SSI pre- and postoperative models had an AUC of 0.76 and 0.85, respectively, compared to leaks with an AUC of 0.79 preoperatively and 0.86 postoperatively (Table 1). NPV and PPV at ~ 80% sensitivity were likewise similar. NPVs remained high ranging from 0.98–0.99. SSI models achieved a PPV of 12% and 19%, leak models 7% and 10%, respectively (Table 1). Overall risk prediction was achieved based on a lower number of features. The preoperative SSI model relied on 12 features, the most important being age, past procedure type, and BMI (Fig. 1a). Postoperative SSI prediction was achieved through 15 input features with the surgical approach (open versus laparoscopic), surgery duration, and age having the largest impact (Fig. 1b). Preoperative leak prediction depended on eight inputs including past procedure type, ASA score, and age (Fig. 1c). Postoperatively, the leak model required ten inputs with open versus laparoscopic surgery, surgery duration, and past procedure type being the most important (Fig. 1d). Further evaluation using SHAP values further reveals how individual features and their values affect the models’ predictions (Fig. 2 for SSI and Fig. 3 for leaks).

The SSI models’ decision boundaries and the direction in which different feature values affect the predictions largely aligned with previously published literature (Fig. 2a,b). As expected, patients aged ~ 55 years or older increase the models’ SSI risk prediction, so do ASA scores larger than 2, low preoperative albumin (< ~ 3.5 g/dL), urgent surgery, more than ~ 7 days preoperative stay, and one or more microbiology tests performed in the three days leading up to surgery. The type of procedure is also an important factor for forecasting postoperative SSI. ‘Small bowel’ and ‘colorectal’ procedure types increased the models’ prediction of SSI substantially, no matter whether they were performed as part of a previous surgery (in the case of the preoperative model, Fig. 2a) or as a past or current surgery (for the postoperative model, Fig. 2b). BMI larger than ~ 30 kg / m2 and smoking decreased the model's risk prediction of SSI in line with the small odds ratios in Table 2. While gender is one of the chosen features for both, pre- and postoperative models, it does not clearly adjust the risk higher or lower suggesting that there are interactions with other variables that affect how it impacts the risk. For preoperative SSI prediction, a diabetes diagnosis and preoperative antibiotics up to ~ 2 h before surgery also elevated the model’s risk score (Fig. 2a). The postoperative SSI model relied strongly on data collected intraoperatively: Open surgery, a surgery duration of more than ~ 2 h, more than one surgical procedure, blood loss larger than 100 mL, and a blood transfusion increased the model’s risk score (Fig. 2b).

Like SSI models, leak prediction models also relied strongly on the type of procedure and followed cutoffs identified in the literature with only a few exceptions (Fig. 3a,b). ‘Small bowel’ and ‘colorectal’ procedures as a past procedure (for pre- and postoperative leak model) or as the current procedure increased the models’ risk prediction for leaks. As identified in the previous literature, ASA score ≥ 2, age ≥ 50 or 60 respectively, total serum blood ≤ 7.8 g/dL preoperatively or 7.2 g/dL postoperatively as well as male gender increased the model’s risk score. BMI ≥ 30 kg / m2 also decreased the models' risk prediction as in the case of SSI, and low albumin had mixed effects. While it increased the risk in the preoperative leak model, albeit with a higher cutoff of 4.4 g/dL, in the postoperative model any albumin value (in contrast to not having albumin measured) appears to increase the model's risk prediction. One of the relevant features for preoperative leak prediction is anticoagulant use. However, its effect on the risk score is unclear, which again may point to some interaction with another factor. As for postoperative SSI models, postoperative leak models also depend on intraoperative data sources. Open surgery, surgery duration longer than 160 min, and blood transfusion increase the model's risk prediction accuracy.

## Discussion

Despite the huge interest in artificial intelligence applications to support clinical-decision making, few examples exist where machine learning models are successfully deployed in clinical practice. Reasons for this discrepancy are multifold, ranging from limited access to large diverse datasets that allow training and validation of generalizable and unbiased algorithms to difficulties in integrating AI systems into clinical workflows^32^. One major obstacle for the adaptation of machine learning algorithms into clinical practice arises from distrust in the decisions made by ‘black box’ models whose inner workings cannot be understood easily^33^. To improve the trust in and the use of machine learning models for improving patient outcomes, it is essential to demystify ‘black box’ models. Here, we use the example of risk assessment for postoperative complications to demonstrate how explainable AI models can be developed that are grounded on medical knowledge, utilize electronic health record data, and allow for non-linear relationships to be captured.

As a first step, we trained Gradient Boosting models, tree-based algorithms that can capture nonlinear relationship, on all data sources available at the prediction time (pre- or postoperative). These models served as baseline models to predict postoperative SSI and leak complications. As previously shown, the integration of intraoperative data in the postoperative models improved the predictive performance compared to the preoperative models^19,34^. The AUC of SSI risk prediction models increased from 0.76 preoperatively to 0.86 postoperative (Table 1). A comparable previous study reported an AUC of 0.74 and 0.75 for pre- and postoperative wound risk prediction models^19^. Other studies that only developed preoperative SSI models achieved a larger AUC of 0.82 when including procedure type as one of the input variables^9,18^. In our case, procedural information was only available postoperatively and was therefore not included in preoperative models. Leak risk prediction models improved their AUC from 0.78 preoperatively to 0.86 postoperatively (Table 1; AUC of 0.75 and 0.81, respectively, if only eligible procedures were considered, Supplementary Table S9). Published leak prediction scores also include intraoperative information and report AUC values of 0.83 and 0.84^35,36^. These scores also include information on the number of hospital beds^35^ and distance of the anastomosis to the anal verge^36^ which likely had a positive effect on predictive performance.

The developed baseline models depend on many features (Table 1), including data sources that have not been identified as risk factors in the literature before. Examples are prothrombin, eternal CO2, or analgesic drugs. This is not surprising as machine learning models rely on patterns and variations in the input data and do not necessarily integrate data features that are causal from a medical perspective. We were interested in whether models based on previously identified risk factors would perform equally well as those incorporating all the input data. As a second step, we, therefore, identified evidence-based pre- and intraoperative risk factors for SSI and leak complications and trained Gradient boosting models on these inputs. These Literature-based models performed similarly well to their baseline, frequently more complex counterparts (Table 1). Since these models solely relied on features available in our EMR dataset, other published risk factors would likely improve the predictions. Examples are wound class and surgical skill for SSI^21,23,37^ and location, size, and stage of the tumor as well as surgical experience for leaks^25,31^.

As a third step, we attempted to explain the inner workings of the literature-based Gradient boosting models by analyzing the contribution of the different features and their values on the predictions using Shap values. Shap analysis revealed that the models attribute higher (or lower) risk to feature-value combinations based on similar cutoffs as well-established thresholds (Figs. 2 and 3). Shap analysis has also been used in a previous study to describe the inner workings of postoperative risk prediction models. Xue et al. trained machine learning models to predict postoperative complications on around 700 features extracted from EMR data^34^. They transformed the SHAP values from all the extracted, frequently abstract features into a clinical feature space that is interpretable by physicians^34^. The advantage of this approach is that data is not preselected before modeling, the disadvantage is that the models may be accidentally fitted to confounders rather than meaningful clinical relations^32^.

Besides explaining the models’ decision-making process, Shap analysis can also be utilized to identify factors that drive an individual patient’s risk and could, therefore, support physicians in defining individual risk mitigation strategies. Patient-specific Shap analysis, automatically performed each time when the risk of the patient is assessed, provides an overview of the patients’ risk factors that increase/decrease the risk and to what degree. Surgeons can utilize this information to either modify risk factors by advising lifestyle changes, e.g. in the case of BMI and albumin (as an indicator of malnutrition), or wherever possible by choosing certain procedural modifications, e.g. in the case of open versus laparoscopic approaches. While the current risk models only have few modifiable risk factors future research will hopefully expand their number and thus make explainability of individual patient risk factors even more valuable. Even in the absence of being able to modify risk factors identification of high-risk patients is valuable. Preventive treatments, too costly to be applied to every patient, or more stringent surveillance to enable early detection and intervention could be specifically applied to high-risk patients.

Notably, all risk prediction models had a high NPV of 0.97–0.99 (Table 1 and Supplementary Table S9). The NPV describes the ratio of correctly identified patients without a complication. Therefore, The models’ predictions are particularly useful to confidently identify patients that most likely will not develop a postoperative complication. In contrast, PPV, describing the ratio of correctly identified patients with a complication, was substantially lower, ranging from 0.12–0.19 (Table 1 for SSI and Supplementary Table S9 for leak eligible cases). This means that one in five to ten patients that are predicted to be at high risk will develop the complication. Depending on the complication, even this low number can provide clinical value. Although preventive strategies or close patient surveillance would have to be applied to five to ten times more patients the benefit for patients whose complications are prevented or attenuated may outweigh the additional workload. Similarly, costs may be reduced if the total costs of all applied prevention strategies are lower than the costs associated with the treatment of the complication.

Surprisingly, BMI larger than ~ 30 kg / m2 and smoking decreased the risk of SSI, as seen in the odds ratios (Table 1) and the explanation of the model’s predictions (Fig. 2). Both, high BMI and smoking have been associated with increased risk of SSI in published literature ^38–41^. Conversely, other studies have found BMI and smoking not to be a consistent risk factors for SSI. Some studies have shown BMI not to impact SSI or outcomes^42,43^. Others report obesity measures other than BMI that are more strongly correlated with outcomes, such as waist circumference^41,44^. Data on smoking is mixed while typically demonstrating it as a risk factor. Some studies have found it not to be a factor in models where it was examined^45^. Interpretation is further conflated by recent data in 55,240 patients examining the relationship between BMI and smoking that showed a stepwise increase in SSI rate as BMI increased, with smoking adding additional risk in each group^46^. While we cannot explain the results for smoking in this data set, the observations for high BMI can be explained by a large fraction of 85% of overweight patients undergoing bariatric surgery. These weight reduction surgeries are associated with high BMI while also having a low risk of SSIs, particularly when performed laparoscopically as in our data set^47,48^. We will continue to evaluate the variables BMI and smoking and their effect on the models’ predictions with ongoing testing and retraining in larger, more diverse populations.

We note that there are limitations to this study. Our dataset consisted of 4 k surgeries performed in a single department of a single institution. The potential problem with small samples is their limited diversity and applicability to other data samples. Since our models have been trained on patients undergoing surgeries in a general and oncology department in Israel, they have learned patient and surgery characteristics from this dataset to predict the risk of SSI and leak complications. However, the risk of complications may manifest differently when other patient populations are considered. Our dataset underrepresents different races, geographical areas, and clinical specialties. A larger, more diverse dataset will be required to improve and validate our models.

The strengths of the presented modeling approach are multifold: (1) The developed risk prediction models rely on well-established risk factors. To base machine learning models on medical knowledge grounded on years of research has the advantage that the model is likely more robust when applied to different datasets. It is also helpful for physicians who are aware of the individual risk factors but may struggle in weighting and combining them into an overall risk estimate, a task that machine learning models excel in when trained on the appropriate data. (2) Our risk predictions are transparent, both in terms of the models' overall decision-making and individual predictions. Being aware of the inner working of the model builds trust in its results and helps physicians define mitigation strategies for reducing risk. (3) The models are based on EMR data. Leveraging available EMR data ensures that the models can be integrated into clinical workflows as seamlessly as possible without adding unnecessary burden to hospital staff by requiring manual data inputs. Going forward, it will be important to test the risk models in clinical settings to validate their performance on current real-world data and to integrate the prediction into the day-to-day workflow of surgeons and staff. The physicians’ interaction with the model output and its acceptance will also have to be investigated^49^. Only if physicians assess the risk more accurately following AI interaction and appropriate mitigation strategies are adopted can machine learning algorithms reduce postoperative complications and improve patients’ health.

## Conclusion

Risk prediction models for postoperative complications do not need to be unexplainable black-box models. Models trained on evidence-based risk factors perform on par with those trained on a larger number of EMR-based inputs. Model explanation using Shap analysis cannot only help build trust in machine learning models but can also support physicians in identifying risk mitigation strategies.

## Supplementary Information

Supplementary Information 1.

Supplementary Information 2.

## Data Availability

The data that support the findings of this study are available from Sheba Medical Center but restrictions apply to the availability of these data, which were used under license for the current study, and so are not publicly available. However, data are available from the authors upon reasonable request and with permission of and through Sheba Medical Center.
