# Supplementary material for: Towards interpretable, medically grounded, EMR-based risk prediction models
Source: Sci Rep. 2022 Jun 15;12:9990. doi: 10.1038/s41598-022-13504-7 (PMC9200841; doi:10.1038/s41598-022-13504-7)
Supplement: Supplementary file 1 — Supplementary Information 1. [file 41598_2022_13504_MOESM1_ESM.pdf]

Supplementary

Towards interpretable, medically grounded, EMR-based risk prediction models

Isabell Twick, Guy Zahavi, Haggai Benvenisti, Ronya Rubinstein, Michael S. Woods, Haim Berkenstadt, Aviram Nissan, Enes Hosgor, Dan Assaf

1. Feature creation

Differentiation between preoperative and intraoperative features

Data points collected from department admission to surgery start were included in preoperative models. For postoperative risk prediction data points recorded from department admission to surgery end were taken into account. Surgery start was defined as the earliest among the timestamps *patients in operating room*, *anesthesia start* and *surgery start*. Accordingly, surgery end was based on the latest of the timestamps *patient out of operating room*, *anesthesia end* and *surgery end*.

Preoperative features included patient demographics, comorbidities, habits, regular drugs and past procedures as well as preoperative measurements. Preoperative measurements contained vitals, blood and microbiology tests as well as anesthesiologist checks. Postoperative risk prediction was based on preoperative features combined with additional data recorded during surgery. Intraoperatively collected features included procedure types, surgery specifics, administered drugs and intraoperative vital and anesthesia measurements. A full list of all features with pre- / postop flag is provided in the additional Supplementary file: *Supplementary - List of features.xlsx*.

Missing data

For categorical features an additional category for missing values was created. For count features missing values were imputed by 0. Other missing values were not imputed and handled by the *CatBoost* algorithm.

Procedure classifications

Procedures and *past procedures* were classified into procedure types. This was achieved by searching all procedures of the surgery of interest and all past procedures performed for the 'search terms' listed in Supplementary Table S1. If the term was present in at least one of the procedures the surgery was labeled with the respective surgery type. In this way features called "procedure colorectal", "procedure small bowel", etc. were created. This classification resulted in surgeries being assigned to more than one procedure type. To define a unique procedure type per surgery the features "procedure type" and "past procedure type" assign the procedure type with the highest priority as shown in Supplementary Table S1 to the surgery. "Intestinal" procedures refer to "colorectal" and "small bowel" procedures.

Supplementary Table S1: Procedure classification

Priority	Procedure type	Search terms
1	Colorectal excl. appendectomy	Rectum, colectomy, colostomy, ileocolic, sigmoidectomy, rectal, proctectomy, colon, mesorectal, proctopexy, large bowel, recto, cecectomy, large intestine, cecostomy
2	Small bowel	Small bowel, ileostomy, jejunostomy, enterolysis, enteroenterostomy, small intestine, meckel, stricturoplasty
3	Abdomen, Retroperitoneum	Adrenalectomy, splenectomy, pancreatectomy, nephrectomy, omentum, intra abdominal, nephroureterectomy, abdominal wall, umbilectomy

4	Gynaecology, pelvic, incl cancer	Cytoreductive, omentectomy, salpingo oophorectomy, hysterectomy, pipac, cystectomy
5	Hepatic	Hepatectomy, liver, hepatico
6	Appendectomy	Appendectomy
7	Hernia	Hernia repair, umbilical hernia, inguinal hernia, parastomal hernia
8	Biliary tract	Cholecystectomy, biliary duct
9	Anorectal	Hemorrhoids, anorectal, anal, anus, perirectal
10	Gastroesophagal	Gastrectomy, gastric, paraesophageal, gastrojejunostomy, gastrostomy, esophagectomy, antrectomy, stomach
11	Breast	Lumpectomy, axillary lymph, axillary sentinel lymph, mastectomy, breast, nipple, axillary node
12	Diagnostic	Diagnostic, examination, exploratory, laparotomy, laparoscopy

Organ and Diagnosis classifications

Organ fields of the surgery of interest and of past procedures were classified into groups. Similarly, *diagnosis at admission to surgery* were grouped. This was achieved by searching the respective data fields for the 'search terms' listed in Supplementary Table S2 and Table S3. If the term was present the surgery was labeled with the respective organ type, e.g. "organ colon" or "organ small intestine", or diagnosis type, e.g. "diagnosis morbid obesity" or diagnosis "carcinoma".

Supplementary Table S2: Organ classification

**Organ type**	**Search terms**
Stomach	gastrect, gastro, gastric
Colon	colon, coelct, colostomy, sigmoidectomy, polypectomy, large bowel, cecectomy
Small intestine	ileostomy, enteroenterostomy, small bowel, Meckel, small intestine
Appendix	append
Anus rectum	protect, perianal, rectum, anus, hermorrhoids, anal, recatl, proctopexy
Pancreas	pancreatect, pancreas
Gallbladder	cholecyst
Hernia	hernia
Peritoneum	cytoreductive, periton, omentum, omentect, epiploect periton
Breast	lumpectomy, mastectomy, breast
Reproductive organs	salpingo-oophorectomy, hysterectomy, vaginal, ovarian
Liver	hepatectomy, liver
Lymph node	lymph node, lymphadenectomy
Kidney	nephrectomy, kidney

Supplementary Table S3: Diagnosis classification

Diagnosis type	Search terms
Morbid obesity	morbid obesity
Carcinoma	carcinoma
Colon carcinoma	colon carcinoma, carcinoma of colon
Breast carcinoma	breast carcinoma, carcinoma of breast
Inguinal hernia	inguinal hernia, hernia inguinal
Hernia	hernia
Appendicitis	appendicitis
Acute appendicitis	acute appendicitis
Choletithiasis	choletithiasis
Melanoma	melanoma
Colon	colon, large bowel
Breast	breast
Acute	acute
Mass	mass
Obstruction	obstruction
Bowel	bowel
Stomach	gastrect, gastro, gastric, stomach
Anus rectum	perianal, rectum, anus, hermorrhoids, anal, rectal
Cholecystitis	cholecystitis

Preoperative Measurements

Preoperative vital measurements (*diastolic pressure, systolic pressure, fever, pain, pulse* and *saturation*) and lab results were summarized by several metrics. For all vitals and labs parameters the raw test results in numeric form were used.

Similar to a recent study (Saria et al., 2013) we thought to capture short- and more long-term measurements. Thus, calculations were based on data from the 24hrs leading up to surgery (referred to as "0 days") and based on data between 96 and 24hrs leading up to surgery (referred to as "-4 to -1 days"). For both of these timeframes the mean was calculated, and the number of measurements counted. Additionally, the most recent measurement prior to surgery (referred to as "last") was included in the features. To further provide some information based on the medical literature we also counted the number of high/normal/low measurements for the two timeframes. For lab results "high"/ "low" measures were determined according to the result classification in the EHR, for vital measurements normal measures were defined as shown in Supplementary Table S4.

Supplementary Table S4: Normal ranges

parameter	minimum	maximum
pulse	50	100
saturation	94	99
Diastolic pressure	60	80
Systolic pressure	90	120

Fever	36.1	37.2
Mean blood pressure	65	115
CO2 response rate	6	36
etCO2	35	45
Anesthetic agent minimal alveolar concentration	0	1
ECG heart rate	50	100
V2, V5, AVL, median ST	-1	2

Intraoperative Measurements

<u>Preprocessing of intraoperative features:</u>

The following data points collected intraoperatively were considered for intraoperative feature creation: *CO2 response rate, etCO2, type of anesthetic agent, anesthetic agent minimal alveolar concentration, anesthetic agent inspiratory concentration, anesthetic agent expiratory concentration, pulse, saturation, diastolic pressure, systolic pressure, mean blood pressure, fever, ECG heart rate, ECG PVC, V2 ST, V5 ST, AVL ST*.

For parameters CO2 response rate, etCO2, type of anesthetic agent and anesthetic agent alveolar concentration measurements that indicated “no measurement” were removed from the data. For parameters pulse, saturation, diastolic pressure, systolic pressure, mean blood pressure, fever, ECG hear rate, ECG PVC, V2 ST, V5 ST and AVL ST outliers were removed. Outliers were defined as values that were more than three standard deviations away from the mean. Mean standard deviations were calculated on the training set.

Following the cleaning steps the following metrics were calculated from the dataset

- Median ST: median measure of V2 ST, V5 ST, AVL ST as the individual measures were prone to error
- Anesthetic agent inspiratory expiratory difference: difference between inspiratory and expiratory anesthetic agent concentration

<u>Comparing Intraoperative measures to preoperative baseline</u>

Intraoperative measurements were compared to their respective preoperative baseline measurements. Baseline measurements were defined as the first measurement after department admission. Baseline comparison was performed for the following parameters: *pulse, saturation, systolic pressure, diastolic pressure, mean blood pressure and fever*. Baseline comparison was calculated as follows: (*intraoperative measurement – preoperative baseline measurement*) / *preoperative baseline measurement* and named *pulse to baseline*, *saturation to baseline*, etc.

<u>Defining abnormal values</u>

If intraoperative measurements deviated more than 20% from preoperative baseline measurement, they were considered abnormally high or low (see paragraph above for baseline comparison). If intraoperative measurements were lower than ‘minimum’ or higher than ‘maximum’ in table (Supplementary Table S4) that displays the normal ranges of the respective parameters, they were likewise considered abnormally low or high.

Feature Calculation:

Mean, standard deviation, minimum and maximum were calculated for the following intraoperative measurements: *CO2 response rate, etCO2, pulse, saturation, diastolic pressure, systolic pressure, mean blood pressure, fever, ECG heart rate, ECG PVC, o2.fi, pulse to baseline, saturation to baseline, systolic pressure to baseline, diastolic pressure to baseline, mean blood pressure to baseline, fever to baseline.*

Additionally, the total number of measurements as well as the number of high or low and normal measurements were counted. The counts of high, low and normal measurements were divided by the total number of measurements to gain the percentage of high/low/normal measurements. These calculations were performed for parameters: *CO2 response rate, etCO2, pulse, saturation, diastolic pressure, systolic pressure, mean blood pressure, fever, ECG heart rate, V2 ST, V5 ST, AVL ST, median ST, pulse to baseline, saturation to baseline, systolic pressure to baseline, diastolic pressure to baseline, mean blood pressure to baseline, fever to baseline.*

For measures *V2 St, V5 ST, AVL ST* the longest monotonously increasing path was calculated (where monotonously increasing was defined as measure at time t >= measure at time t-e). Further, max ST was calculated as the maximum of *V2 ST, V5 ST and AVL ST*, as well as the maximum ST increase as the maximum length of monotonously increasing path of *V2 ST, V5 ST and AVL ST*.

With respect to the anesthetic agents used in surgery the number of anesthetic agents used was counted as well as the number of measurements for every anesthetic used. From the measurements counts of an anesthetic agent, the percentage of that agent with respect to all anesthetic agents' measurements were calculated.

Finally, two features describing the length of surgery were computed: *Intraoperative measures length minutes* describes the length of the first to the last intraoperative measure. *Surgery length minutes* refers to the time between surgery start and surgery end.

Outcomes

Patient files were reviewed by surgeons prior to patient discharge and complications manually recorded according to the Clavien Dindo complication scale. Manually annotated complications were merged to surgeries based on patient id and date the complication was reported. Cases where patients underwent several surgeries were reviewed separately to ensure that the complications were assigned to the correct surgery.

2. Cohort Summary

Supplementary Table S5: Population characteristics

**General**	
Number of surgeries, n	**4004**
Number of patients, n	3440
**Demographics**	
Female Gender, n (%)	2182 (54.5)
Age, median (25th -75th)	55 (40 – 67)
**Comorbidities**	
Number of diseases, median (25th – 75th)	0 (0 – 1)
Neoplasms, n (%)	789 (19.7)
Circulatory Diseases, n (%)	357 (8.9)
Metabolic Diseases, n (%)	336 (8.4)
Digestive Diseases, n (%)	295 (7.4)
Mental Diseases	93 (2.3)
Tissue Diseases, n (%)	92 (2.3)
Genitourinary Diseases, n (%)	79 (2.0
Nervous System Diseases, n (%)	70 (1.7)
Eye Diseases, n (%)	59 (1.5)
Respiratory Diseases, n (%)	43 (1.1)
**Medications**	
Number of medications, median (25th – 75th)	1 (0 – 4)
Cardiovascular Drugs, n (%)	1437 (35.1)
Metabolism Drugs, n (%)	1327 (33.1)
Blood Drugs, n (%)	866 (21.6)
Nervous System Drugs, n (%)	865 (21.6)
Hormonal Drugs, n (%)	365 (9.1)
Respiratory Drugs, n (%)	281 (7)
Reproductive Urinary Drugs, n (%)	216 (5.4)
**Operative Characteristics**	
Laparoscopic surgery	1876 (46.9)
Urgent surgery	1012 (25.3)
Surgery Type	
Hernia, n (%)	576 (14.4)
Gastroesophageal, n (%)	529 (13.2)
Colorectal, n (%)	513 (12.8)
Biliary tract, n (%)	449 (11.2)
Breast, n (%)	402 (10)
Diagnostic, n (%)	366 (9.1)
Appendectomy, n (%)	333 (8.3)
Small bowel, n (%)	314 (7.8)
Abdomen / Retroperitoneum, n (%)	102 (2.5)
Anorectal, n (%)	96 (2.4)
Gynecological	64 (1.6)
Hepatic	37 (0.9)
**Outcomes**	
ICU admission, n (%)	109 (2.7)
Length of stay, median (25th – 75th)	2 (1 – 7)

30-day readmission, n (%)	57 (1.4)
30-day mortality, n (%)	51 (1.4)
**Surgical Site Infection (SSI), n (%)**	**234 (5.8)**
SSI superficial, n (%)	201 (5)
SSI deep, n (%)	22 (0.5)
SSI organ space abscess	11 (0.3)
**Leaks**	**137 (3.4)**
Gastrointestinal leak	119 (2.9)
Biliary leak	6 (0.1)
Pancreatic leak	3 (0.1)
Anastomosis leak	1 (0.1)
Not defined	8 (0.2)

Supplementary Table S6: Counts of SSI and Leaks by surgery type

	**Total, n (%)**	**SSI, n (%)**	**Leak, n (%)**
**All**	4004 (100)	234 (5.8)	137 (3.4)
**Surgery Type**			
Hernia	576 (14.4)	11 (1.9)	3 (0.5)
Gastroesophageal	529 (13.2)	4 (0.8)	10 (1.9)
Colorectal	513 (12.8)	95 (18.2)	48 (9.1)
Biliary tract	449 (11.2)	5 (1.1)	8 (1.8)
Breast	402 (10)	3 (0.7)	0 (0)
Diagnostic	366 (9.1)	27 (7.3)	17 (4.6)
Appendectomy	333 (8.3)	7 (2.1)	2 (0.6)
Small bowel	314 (7.8)	56 (17.6)	40 (12.4)
Abdomen / Retroperitoneum	102 (2.5)	11 (10.7)	7 (6.7)
Anorectal	96 (2.4)	1 (1)	0 (0)
Gynecological	64 (1.6)	4 (6.2)	1 (1.6)
Hepatic	37 (0.9)	1 (2.7)	1 (2.7)

3. Model Performance

Supplementary Table S7: Model performances

Table shows AUC performance values for the different SSI and leak risk prediction models. The mean AUC and its 95% Confidence Interval of 5-fold cross validation (CV) experiments performed on the training set is displayed as well as the AUC of the final models that were retrained on the whole training set and the associated AUC of the test set.

Model	Pre / Post	Features	Mean AUC 5-fold CV train	95% CI 5-fold CV train	AUC whole train	AUC test
SSI models						
**Naïve Gradient Boosting**	Pre	8	0.748	(0.716 - 0.780)	0.784	0.758
	Post	56	0.847	(0.815 - 0.879)	0.914	0.857
**Literature-based Gradient Boosting**	Pre	12	0.750	(0.741 - 0.759)	0.836	0.762
	Post	15	0.845	(0.820 - 0.870)	0.892	0.853
Leak models						
**Naïve Gradient Boosting**	Pre	60	0.770	(0.745 - 0.795)	0.785	0.775
	Post	82	0.833	(0.799 - 0.867)	0.891	0.855
**Literature-based Gradient Boosting**	Pre	8	0.740	(0.714 - 0.766)	0.877	0.793
	Post	10	0.840	(0.827 - 0.853)	0.917	0.860

Supplementary Table S8: High/low risk group classification using MCC

Table shows classification of patients into high and low risk for the different SSI and leak risk prediction models when the probability threshold is chosen according to the largest Matthew Correlation Coefficient (MCC). MCC, Sensitivity, Specificity, positive predicted value (PPV), and negative predicted value (NPV) with respect to the determined probability threshold are shown.

Model	Pre/ Post	Threshold	MCC	Sensitivity	Specificity	PPV	NPV
SSI models							
**Naïve Gradient Boosting**	Pre	0.060	0.236	0.506	0.855	0.191	0.962
	Post	0.048	0.345	0.779	0.812	0.219	0.982
**Literature-based Gradient Boosting**	Pre	0.062	0.222	0.455	0.871	0.192	0.959
	Post	0.052	0.339	0.792	0.800	0.211	0.983
Leak models							
**Naïve**	Pre	0.037	0.186	0.487	0.875	0.114	0.981
	Post	0.035	0.276	0.744	0.847	0.139	0.990

**Gradient Boosting**							
**Literature-based Gradient Boosting**	Pre	0.032	0.186	0.744	0.732	0.084	0.989
	Post	0.037	0.259	0.744	0.831	0.127	0.991

Supplementary Table S9: Model performances only considering leak eligible cases

Table depicts model performance of all pre- and postoperative leak models developed as part of this study when only considering leak eligible cases. To calculate performance metrics surgeries with the following procedure types were included: “gastroesophageal”, “biliary tract”, “colorectal” and “small bowel”. Table contains AUC (area under the ROC curve) computed for the test set as well as specificity, PPV (positive predicted value), NPV (negative predicted value) with respect to the probability threshold chosen to achieve 80% sensitivity.

Model	Pre/ Post	Features	AUC	Threshold	MCC	Sensitivity	Specificity	PPV	NPV
Leak models									
**Naïve Gradient Boosting**	Pre	60	0.749	0.033	0.160	0.788	0.542	0.104	0.974
	Post	82	0.812	0.031	0.229	0.788	0.666	0.137	0.979
**Literature-based Gradient Boosting**	Pre	8	0.769	0.031	0.201	0.788	0.619	0.122	0.977
	Post	10	0.813	0.035	0.251	0.788	0.699	0.149	0.980

4. Naïve Gradient Boosting Models

Supplementary Figure S1: "SSI Naïve Gradient Boosting"

Prediction models for SSI risk assessment based on all pre-calculated features combined with algorithmic feature selection: (A) Preoperative SSI risk model based on data collected before surgery start. (B) Postoperative SSI risk model based on data collected up to surgery end. Top: SHAP summary plot depicting how the feature values affect the prediction. Bottom: Table containing feature explanations. (SHAP plot of postoperative model only shows the twenty most important features.)

a) Preoperatively

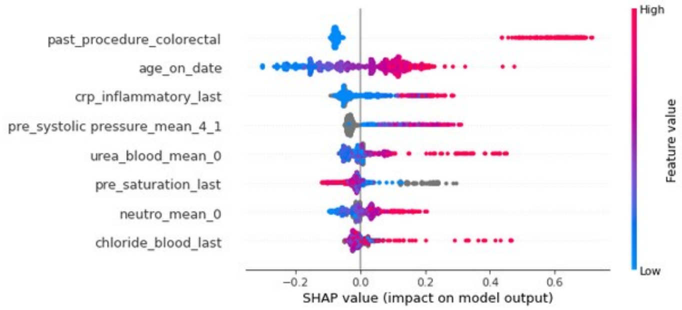

Feature name	Explanation
Past procedure colorectal	Previous colorectal surgery
Age	Age
CRP inflammatory last	Most recent preoperative CRP inflammatory result
Pre systolic pressure mean 41	Average systolic pressure -4 to -1 days before surgery
Urea blood mean 0	Average urea blood in the 24hrs up to surgery
Pre saturation last	Most recent preoperative oxygen saturation
Neutro mean 0	Average neutro result in the 24hrs before surgery
Chloride blood last	Most recent chloride blood result

b) Postoperatively

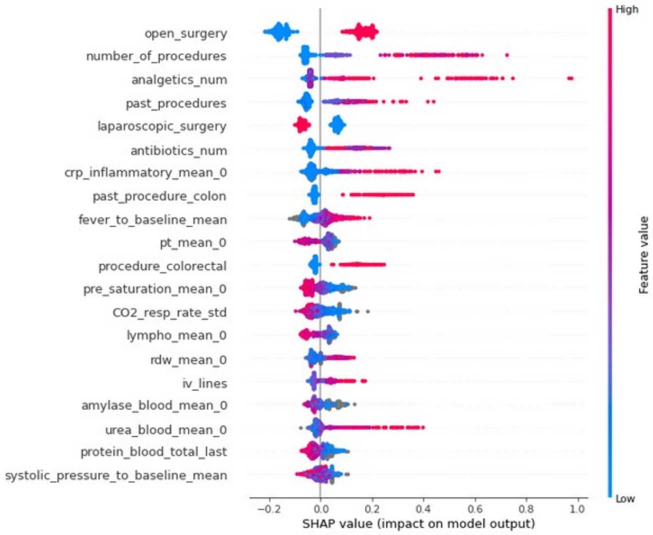

Feature name	Explanation
Open surgery	Whether the surgery was open (versus laparoscopic)
Number of procedures	Number of procedures performed during surgery
Analgetics num	Number of alangetics given in surgery
Past procedures	Number of past procedures
Laparoscopic surgery	Whether the surgery was laparoscopic (versus open)
Antibiotics num	Number of antibiotics given in surgery
CRP inflammatory mean 0	Average CRP inflammatory in the 24hrs priot to surgery
Past procedure colon	Whether the patient had a colon surgery in the past
Fever to baseline mean	Average difference between intraoperative temperature and preoperative baseline
Pt mean 0	Average pt result in the 24hrs before surgery
Procedure colorectal	Colorectal procedure
Pre saturation mean 0	Average saturation in the 24hrs before surgery
CO2 resp rate std	Standard deviation CO2 respones rate during surgery
Lympho mean 0	Average Lympho in the 24hrs before surgery
RDW mean 0	Average RDW in the 24hrs before surgery
IV lines	Number of IV lines
Amylase blood mean 0	Average amylase blood in the 24hrs before surgery
Urea blood mean 0	Average urea blood in the 24hrs before surgery
Protein blood total last	Most recent protein blood total
Systolic pressure to baseline mean	Average difference between intraoperative systolic pressure and preoperative baseline

Supplementary Figure S2: “Leaks Naïve Gradient Boosting”

Prediction models for leaks risk assessment based on all pre-calculated features combined with algorithmic feature selection: (A) Preoperative SSI risk model based on data collected before surgery start. (B) Postoperative SSI risk model based on data collected up to surgery end. Top: SHAP summary plot depicting how the feature values affect the prediction. Bottom: Table containing feature explanations. (SHAP plots only show the twenty most important features.)

a) Preoperative Model

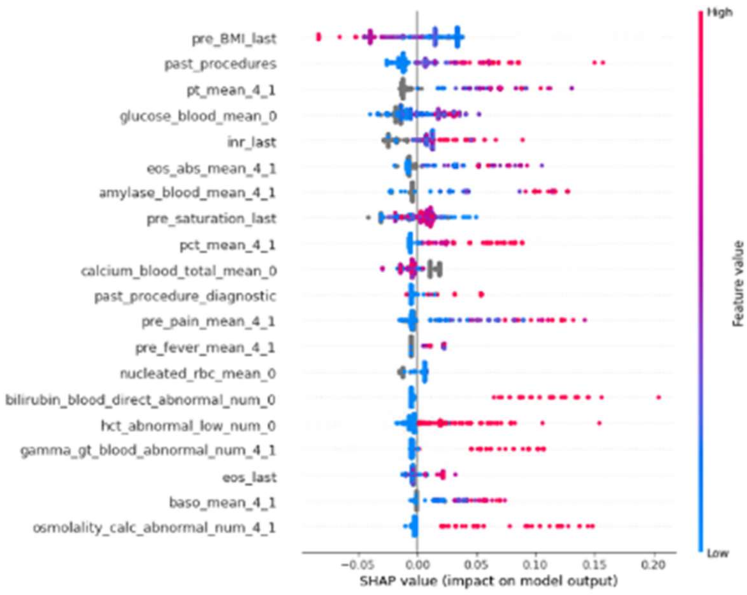

Feature name	Explanation
BMI	Most recent body mass index
Past procedures	Number of previous procedures
Pt mean day 4 to 1	Average pt result on day -4 to -1 before surgery
INR last	Most recent INR measurement
Eos abs mean day 4 to 1	Average EOS absolute on day -4 to -1 before surgery
Amylase blood mean 4 to 1	Average amylase blood on day -4 to -1 before surgery
Pre saturation last	Most recent preoperative oxygene saturation
Pct mean 4 to 1	Average pct measured on day -4 to -1 before surgery
Calcium blood total mean 0	Average calcium blood result in the 24hrs before surgery
Past procedure diagnostic	Previous diagnostic procedure
Pre pain mean 4 to 1	Average pain on day -4 to -1 before surgery
Pre fever mean 4 to 1	Average temperature on day -4 to -1 before surgery
Pre pulse num -4 to -1	Number of pulse measures on day -4 to -1
Nucleated rbc mean 0	Average nucleated rbc in the 24hrs before surgery
Bilirubin blood direct abnormal num 0	Number of abnormal bilirubin blood direct results in the 24hrs before surgery
Hct abnormal low num 0	Number of abnormally low Gamma gt blood in the 24hrs before surgery
Gamma gt blood abnormal num 4 to 1	Number of abnormal gamma gt blood on day -4 to -1 before surgery
Eos last	Most recent eos result
Baso mean 4 to 1	Average baso on day -4 to -1 before surgery
Osmolarity calc abnormal num 4 to 1	Number of abnormal osmolarity calc on day -4 to -1 before surgery

b) Postoperative Model

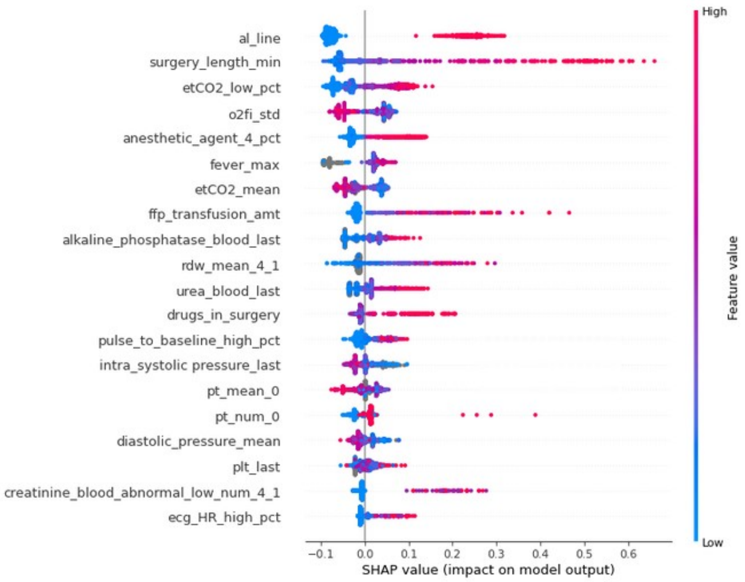

Feature name	Explanation
Al line	Whether artery line in surgery
Surgery length minutes	Surgery duration in minutes
etCO2 low pct	Percentage of low etCO2 during surgery
O2.fi std	Standard deviation of O2.fi during surgery
Anesthesia agent 4 pct	Percentage of anesthesia agent 4 during surgery
Fever max	Maximum temperature during surgery
etCO2 mean	Average etCO2 during surgery
Ffp transfusion amt	FFP (plasma) transfusion amount

Alkaline phosphatase blood last	Most recent alkaline phosphatase blood before surgery
RDW mean 4 1	Average RDW on day -4 to -1 before surgery
Urea blood last	Most recent urea blood before surgery
Drugs in surgery	Number of drugs administered in surgery
Pulse to baseline high pct	Percentage of high difference in intraoperative pulse to preoperative baseline
Intra systolic pressure last	Last intraoperative systolic pressure
Pt mean 0	Average pt in the 24hrs before surgery
Pt num 0	Number of pt measures in the 24hrs before surgery
Diastolic pressure mean	Average intraoperative diastolic pressure
Plt last	
Creatinine blood abnormal low num 4 1	Number of abnormally low creatinine blood measures on days -4 to -1 before surgery
ECG HR high pct	Percentage of high ECG herat rate during surgery
